# High neutrophil-to-lymphocyte ratio is a significant predictor of depressive symptoms in maintenance hemodialysis patients: a cross-sectional study

**DOI:** 10.1186/s12888-022-03963-7

**Published:** 2022-05-03

**Authors:** Jianan Feng, Xiangxue Lu, Han Li, Shixiang Wang

**Affiliations:** Department of Blood Purification, Beijing Chao-Yang Hospital, Capital Medical University, No. 8 Gongti South Road, Chaoyang District, Beijing, 100020 China

**Keywords:** Maintenance hemodialysis, Neutrophil-to-lymphocyte ratio, Depressive symptoms, Inflammation, Patient Health Questionnaire-9

## Abstract

**Background:**

Depression is one of the most important psychiatric disorders in chronic kidney disease patients who undergo maintenance hemodialysis (MHD). Previous studies have shown that low-grade inflammation is involved in the progression of depressive symptoms. The neutrophil-to-lymphocyte ratio (NLR) is an inflammatory marker that is inexpensive and easy to measure. However, the association between NLR and depression symptoms in MHD patients has not been examined.

**Methods:**

In this single-center, cross-sectional study, we included 160 patients undergoing MHD. The Patient Health Questionnaire-9 (PHQ-9) was used to assess depressive symptoms. NLR was calculated as the ratio of neutrophils to lymphocytes. Multinomial logistic regression and multivariate linear regression analyses were used to examine the association between NLR and depressive symptoms in MHD patients.

**Results:**

Depressive symptoms were detected in 36.7% of the 160 MHD patients. Multinomial logistic regression showed that NLR was a significant predictor of mild (odds ratio [OR]: 1.383, 95% confidence interval [CI]: 1.015–1.884, *p* = 0.04) and moderate/moderately severe depressive symptoms (OR: 1.441, 95% CI: 1.017–2.042, *p* = 0.04) in MHD patients, adjusted for age, sex, Kt/V, dialysis duration, history of kidney transplantation, history of hypertension, and Charlson comorbidity index score. In addition, multivariate linear regression analysis showed that NLR was an independent influencing factor for PHQ-9 score in MHD patients, after adjusting for confounding factors.

**Conclusions:**

These findings suggest that NLR can be used as a biomarker for predicting depressive symptoms in MHD patients.

## Background

Depression is one of the most common and serious psychiatric disorders in patients with chronic kidney disease (CKD) and end-stage renal disease (ESRD) that seriously affects the quality of life of patients [1]. A meta-analysis showed that the prevalence of interview-based depression in patients with stage 1–5 CKD and ESRD patients was 21.4% and 22.8%, respectively; moreover, when using self- or clinician-administered rating scales, the prevalence of depressive symptoms in these groups of patients was 26.5% and 39.3%, respectively [2]. Ravaghi et al. [3] found that the prevalence of depression among MHD patients in Iran was estimated to be 62%, and a South Korean study reported that moderate to severe depression is common in MHD patients and is associated with health-related quality of life [4]. Depression increases the rate of hospital admissions, the duration of hospitalization, and the risk of death in patients undergoing MHD [5–7].

Previous studies have shown that inflammation is closely related to the progression of depressive symptoms, and an elevated level of peripheral proinflammatory cytokines in depressed patients is associated with the severity of depression [8]. Persistent low-grade inflammation is characteristic of patients undergoing MHD [9], which suggests that systemic inflammation is a pathomechanism underlying depression in MHD patients. The neutrophil-to-lymphocyte ratio (NLR) is a cheap and accessible indicator of inflammation. Li et al. [10] reported that high NLR is an independent predictor of all-cause and cardiovascular mortality in MHD patients. Furthermore, studies have shown that NLR is associated with a variety of depression states, such as post-stroke depression [11], adolescent depression [12], and severe depression [13].

However, the association between NLR and depressive symptoms in MHD patients has not been explored to date. The Patient Health Questionnaire-9 (PHQ-9) is a reliable and valid screening instrument for assessing depressive symptoms with high overall sensitivity and specificity [14], and it has been validated for screening purposes in dialysis patients [15]. Therefore, we conducted a single-center cross-sectional study to examine the relationship between NLR and depressive symptoms and investigate the prognostic value of NLR in MHD patients.

## Methods

### Patients and study design

**Fig. 1:**
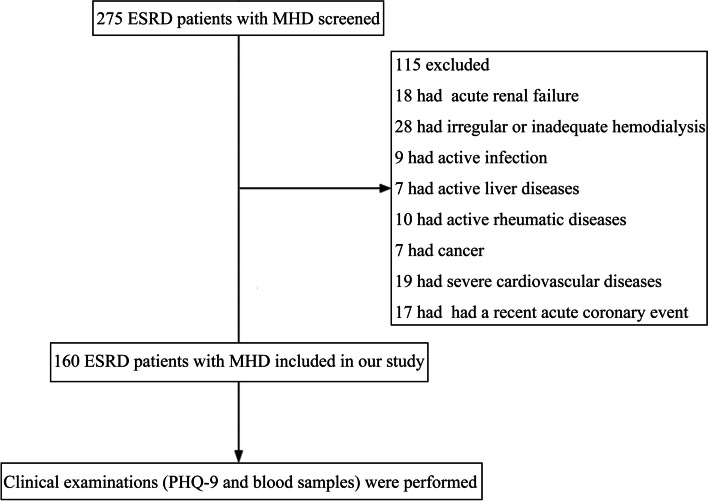
Study flow chart

A total of 160 ESRD patients undergoing MHD who were admitted to the Department of Blood Purification, Beijing Chao-Yang Hospital, Capital Medical University were recruited from July 1, 2020, to July 31, 2020. The inclusion criteria were as follows: (1) ESRD patients who had undergone regular dialysis treatment, (2) aged > 18 years, (3) hemodialysis treatment duration of > 3 months, and (4) in a stable condition. Exclusion criteria were as follows: (1) acute renal failure, (2) infectious diseases within 1 month, (3) active liver diseases or cancer, (4) active rheumatic diseases, (5) irregular or inadequate hemodialysis, and (6) severe cardiovascular or cerebrovascular diseases. The flow chart of the study is shown in Fig. 1.

The dialysis treatment was administered as previously described. ESRD patients underwent hemodialysis three times a week for 4 h per session. Sugar-free bicarbonate dialysates and heparin anticoagulants were used during hemodialysis. Dialysate flow was 500 mL/min, and blood flow was 200–350 mL/min. Dialysate ingredients were 138–140 mmol/L sodium, 2.0–2.5 mmol/L potassium, 1.25–1.5 mmol/L calcium, and 0.5 mmol/L magnesium. The study was conducted in accordance with the Declaration of Helsinki and was approved by the Ethics Committee of Beijing Chao-Yang Hospital, Capital Medical University. Written informed consent was obtained from all participants.

### Data collection

The demographic data collected from patients included age, gender, education level, dialysis duration, pre-dialysis blood pressure, dry weight, ultrafiltration volume, primary cause of renal disease, vascular access type, history of hypertension, and Charlson comorbidity index score.

Fasting blood samples were obtained before dialysis during the mid-week session, and blood cell counts were detected using an automatic hemocyte analyzer (SYSMEX XN-10, Japan). Albumin (Alb), total cholesterol (CHO), triglyceride (TG), serum creatinine (Scr), and blood urea nitrogen (BUN) were detected using an automatic biochemical analyzer (Simens Advia 2400, USA).

### Assessment of depressive symptom status

The PHQ-9 was used to evaluate the depressive symptoms of MHD patients [16]. The PHQ-9 comprises nine items, which are each scored from 0 (not at all) to 3 (nearly every day), with a total score for the nine items ranging from 0 to 27. Scores of 5, 10, 15, and 20 represent cutoff points for mild, moderate, moderately severe, and severe depression, respectively [17].

Participants were allocated to the following groups according to their PHQ-9 scores: (1) no depressive symptoms group for PHQ-9 score of < 5 (*n* = 122), (2) mild depressive symptoms group for PHQ-9 of 5–9 (*n* = 26), and (3) moderate/moderately severe depressive symptoms group for PHQ-9 score of 10–20 (*n* = 12).

### Statistical methods

Sample size was calculated using G-Power version 3.1. For one-way analysis of variance, 159 participants were needed for an estimated medium effect size of 0.25 (f = 0.25), an alpha factor of 0.25 (α = 0.05), and 80% power. Thus, we included 160 patients in the final sample. SPSS version 23.0 (IBM Corp, Armonk, NY, USA) was used to analyze the data. Normally distributed data are expressed as means ± standard deviations. One-way analyses of variance were used to compare multiple groups, and the Bonferroni test was used for post hoc tests between groups. Non-normally distributed data are expressed as medians and interquartile ranges. The Kruskal–Wallis test was used to compare these variables between groups. Categorical variables are expressed as frequencies (%), and ratios were compared between groups using a chi-square test. Multinomial logistic regression was used to identify risk factors for depressive symptoms in MHD patients. In addition, Spearman’s correlation was used for the univariate analysis, and multiple linear regression was used to identify independent influencing factors for depressive symptoms. A *p*-value < 0.05 was considered statistically significant.

## Results

### Demographic and biochemical parameters of patients with varying levels of depressive symptoms

A total of 160 MHD patients were enrolled (mean age 55.5 ± 13.1 years), comprising 90 males (56.2%) and 70 females (43.8%). The median duration of dialysis was 73.5 months (interquartile range 29.3–150.8 months; range 3–343 months). The cause of CKD was chronic glomerulonephritis in 84 patients (52.5%), hypertensive nephrosclerosis in 24 patients (15.0%), diabetic nephropathy in 23 patients (14.4%), polycystic kidney in six patients (3.8%), renal tumor in three patients (7.6%), Henoch-Schonlein purpura nephritis in two patients (1.2%), lupus nephritis in two patients (1.2%), Alport syndrome in one patient (0.6%), and other renal diseases in eight patients (5.0%).

All patients were divided into three groups according to PHQ-9 score: no depressive symptoms group (*n* = 122), mild depressive symptoms group (*n* = 26), and moderate/moderately severe depressive symptoms group (*n* = 12). Table 1 shows that hemoglobin (HGB) and Alb levels in MHD patients with no depressive symptoms and mild depressive symptoms were higher than those of MHD patients in the moderate/moderately severe depressive symptoms group. NLR was lower in the no depressive symptoms and mild depressive symptoms groups than that in the moderate/moderately severe depressive symptoms group. Red cell distribution width (RDW) in the mild depressive symptoms group was higher than that in the no depressive symptoms group. No significant differences were observed between the other parameters shown in Table 1.

**Table 1:** Demographic and biochemical parameters of MHD patients

Item	Total	No Depressive Symptoms (*n* = 122)	Mild Depressive Symptoms (*n* = 26)	Moderate/Moderately Severe Depressive Symptoms (*n* = 12)	F/χ^2^	*P*
Male, n (%)	90 (56.2%)	68 (55.7%)	14 (53.9%)	8 (66.7%)	0.603	0.740
Age (years)	55.5 ± 13.1	55.12 ± 12.3	56.62 ± 14.12	56.67 ± 19.27	0.189	0.828
Dialysis Duration (months)	73.5 (29.3, 150.8)	80 (30, 140)	58 (33, 168)	51 (19, 165)	1.349	0.510
SBP (mmHg)	144.8 ± 21.3	145.2 ± 20.9	144.2 ± 22.4	140.8 ± 25.0	0.241	0.786
DBP (mmHg)	81.3 ± 12.7	81.1 ± 12.6	82.6 ± 14.0	80.4 ± 11.3	0.176	0.839
Kt/V	1.32 ± 0.15	1.32 ± 0.14	1.29 ± 0.13	1.36 ± 0.19	1.17	0.313
Diabetic Mellitus (%)	23 (14.38%)	17 (13.93%)	4 (15.38%)	2 (16.5%)	0.148	0.105
Hypertension (%)	24 (15.0%)	18 (14.75%)	4 (15.38%)	2 (16.5%)	0.138	0.995
Transplantation, n (%)						
Yes	18 (11.3%)	18 (14.75%)	4 (15.38%)	2 (16.5%)	0.138	0.995
No	142 (88.7%)					
WBC (*10^9^/L)	5.99 ± 1.73	5.90 ± 1.67	6.58 ± 2.12	5.72 ± 1.13	1.841	0.162
NEUT (*10^9^/L)	4.04 ± 1.37	3.93 ± 1.26	4.50 ± 1.88	4.08 ± 0.98	1.876	0.157
LY (*10^9^/L)	1.23 ± 0.48	1.23 ± 0.48	1.26 ± 0.39	1.11 ± 0.63	0.435	0.648
HGB (g/L)	114.96 ± 13.63	115.70 ± 12.84^b^	116.15 ± 16.29^c^	104.75 ± 12.02	3.778	0.025^*^
RDW (%)	14.14 ± 2.34	13.92 ± 1.77^a^	15.16 ± 4.15	14.20 ± 1.60	3.096	0.048^*^
PLT(*10^9^/L)	184.32 ± 63.68	181.34 ± 59.70	201.04 ± 82.85	178.42 ± 54.89	1.083	0.341
NLR	3.74 ± 2.06	3.51 ± 1.42^b^	3.85 ± 1.78^c^	5.74 ± 5.17	6.947	0.001^*^
PLR	153.77 (120.05–199.50)	151.95 (122.64–191.21)	169.47 (125.45–207.48)	197.20 (103.64–232.38)	1.374	0.503
Alb(g/L)	42.02 ± 3.24	42.29 ± 2.89^b^	42.16 ± 3.03^c^	39.06 ± 5.31	5.793	0.004^*^
BUN (mmol/L)	22.8 ± 4.81	22.75 ± 4.48	23.07 ± 5.93	22.67 ± 5.76	0.051	0.951
Scr (umol/L)	859.91 ± 226.93	878.16 ± 213.29	834.16 ± 244.94	730.08 ± 289.72	2.576	0.079
BUA (umol/L)	431.19 ± 74.50	432.71 ± 71.68	444.88 ± 78.28	386.08 ± 84.12	2.722	0.069
CHO (mmol/L)	4.17 ± 1.04	4.22 ± 1.03	3.95 ± 1.16	4.10 ± 0.78	0.720	0.489
TG (mmol/L)	1.73 (1.15–2.65)	1.81 (1.20–2.65)	1.55 (1.15–2.82)	1.64 (0.95–2.88)	1.155	0.561
Ferritin (ng/ml)	160.5 (75.8–285.9)	156.5 (75.0–286.1)	189.9 (76.6–267.4)	160.8 (66.6–300.0)	0.205	0.903
hsCRP (mg/L)	3.67 (1.69–7.75)	3.45 (1.67–7.15)	3.67 (2.12–7.44)	8.21 (0.58–13.15)	0.947	0.623
Charlson Comorbidity Index Score	4.34 ± 1.75	4.28 ± 1.69	4.54 ± 1.68	4.58 ± 2.50	0.354	0.702

^a^*p*: no depressive symptoms group vs mild depressive symptoms group

^b^ *p*: no depressive symptoms group vs moderate/moderately severe depressive symptoms group

^c^*p*: mild depressive symptoms group vs moderate/moderately severe depressive symptoms group

^*^Significant at *p* < 0.05

Values are means ± standard deviations or medians (25th–75th percentiles), unless otherwise specified

*Abbreviations*: *SBP* Systolic blood pressure, *DBP* Diastolic blood pressure, *WBC* White blood cell, *NEUT* Neutrophil, *LY* lymphocyte, *HGB* Hemoglobin, *RDW* Red cell distribution width, *PLT* Platelet, *NLR* Neutrophil-to-lymphocyte ratio, *PLR* Platelet-to-lymphocyte ratio, *Alb* Albumin, *BUN* Blood urea nitrogen, *Scr* Serum creatinine, *UA* Uric acid, *CHO* Total cholesterol, *TG* Triglyceride, *hsCRP* High-sensitivity C-reactive protein

### Multivariable logistic analysis between PHQ-9 score and NLR in MHD patients

Table 2 shows the results of the multivariable logistic analysis to identify predictors for mild and moderate/moderately severe depressive symptoms. RDW (OR: 1.216, 95% CI: 1.017–1.455, *p* = 0.032) and NLR (OR: 1.383, 95% CI: 1.015–1.884, *p* = 0.04) were independently associated with mild depressive symptoms, after adjusting for confounding factors. In addition, NLR (OR: 1.441, 95% CI: 1.017–2.042, *p* = 0.04) was independently associated with moderate/moderately severe depressive symptoms, after adjusting for confounding factors.

**Table 2:** Multivariable logistic analysis between PHQ-9 score and NLR in MHD patients

	Mild Depressive Symptoms^a^				Moderate/Moderately Severe Depressive Symptoms^a^			
	Model 1		Model 2		Model 1		Model 2	
	OR (95% CI)	*P*	OR (95% CI)	*P*	OR (95% CI)	*P*	OR (95% CI)	*P*
HGB (g/L)	1.201 (0.986–1.058)	0.245	1.022 (0.985–1.059)	0.246	0.968 (0.922–1.016)	0.184	0.962 (0.910–1.018)	0.910
RDW (%)	1.204 (1.012–1.431)	0.036	1.216 (1.017–1.455)	0.032^*^	0.984 (0.650–1.489)	0.940	0.942 (0.570–1.557)	0.815
NLR	1.398 (1.028–1.900)	0.033	1.383 (1.015–1.884)	0.040^*^	1.451 (1.038–2.028)	0.029	1.441 (1.017–2.042)	0.040^*^
Alb (g/L)	1.062 (0.906–1.244)	0.459	1.083 (0.907–1.294)	0.378	0.815 (0.671:0.990)	0.039	0.800 (0.640–1.001)	0.051

^a^Compared with the no depressive symptoms group

^*^Significant at *p* < 0.05

Model 1: Crude analysis

Model 2: After adjusting for age, sex, Kt/V, dialysis duration, history of kidney transplantation, history of hypertension, and Charlson comorbidity index score

*Abbreviations*: *HGB* Hemoglobin, *RDW* Red cell distribution width, *NLR* Neutrophil-to-lymphocyte ratio, *Alb* Albumin, *CI* Confidence interval, *OR* Odds ratio

### Sensitivity and specificity of NLR in predicting of depressive symptoms by receiver operating characteristic curve analysis

**Fig. 2:**
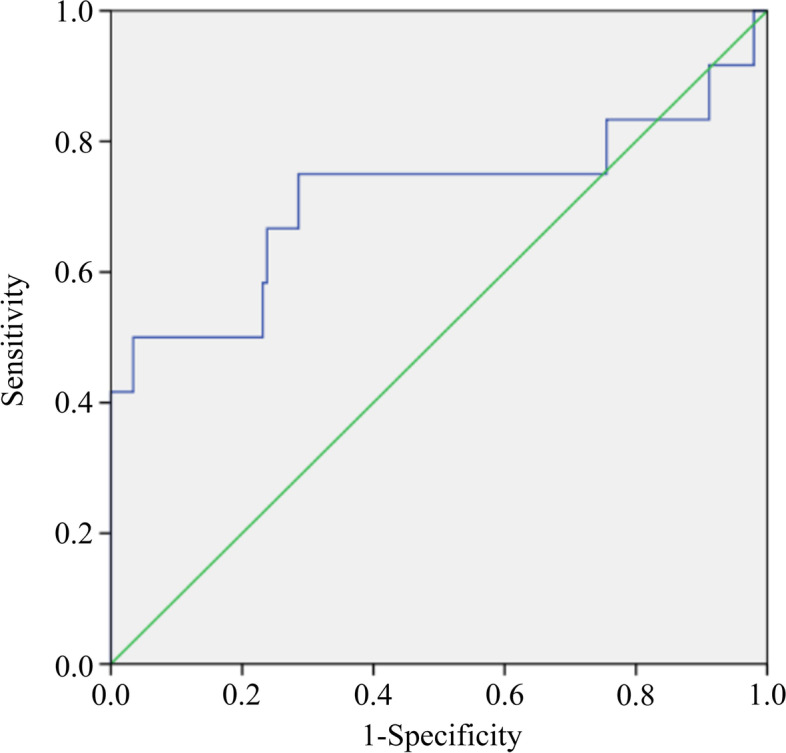
Receiver operating characteristic curves for the prediction of depressive symptoms using NLR

The cutoff value of NLR determined by the receiver operating characteristic (ROC) curve analysis was 4.2 (area under the curve [AUC]: 0.714; 95% CI: 0.508–0.920; 75% sensitivity; 71.4% specificity). Figure 2 shows the ROC curves for the prediction of depressive symptoms using NLR.

### Correlation between NLR and PHQ-9 score in MHD patients

In the univariate analysis, PHQ-9 scores were positively correlated with NLR (*r* = 0.279; *p* < 0.001) and negatively correlated with Alb (*r* =  − 0.209; *p* = 0.008). There was no correlation between PHQ-9 score and gender, age, dialysis duration, diabetes, hypertension, RDW, platelet-to-lymphocyte ratio (PLR), ferritin, HGB, or high-sensitivity C-reactive protein (hsCRP), as shown in Table 3. Furthermore, in the multivariate analysis, the model adjusted for demographic and clinical covariates (i.e., age, gender, Kt/V, dialysis duration, history of kidney transplantation, history of hypertension, and Charlson comorbidity index score) showed that NLR (β = 0.246, 95% CI: 0.175–0.799) was an independent predictor of PHQ-9 score (Table 4).

**Table 3:** Correlation coefficients between PHQ-9 score and NLR and other variables in MHD patients

Variables	*r*	*P*
Male, n (%)	− 0.043	0.587
Age (years)	− 0.036	0.655
Dialysis Duration (months)	− 0.115	0.146
Diabetic Mellitus (%)	− 0.009	0.908
Hypertension (%)	− 0.110	0.166
RDW (%)	0.127	0.110
NLR	0.279	< 0.001^*^
PLR	0.062	0.438
HGB (g/L)	− 0.067	0.398
Alb (g/L)	− 0.209	0.008^*^
Ferritin (ng/ml)	− 0.096	0.225
hsCRP (mg/L)	− 0.006	0.948

^*^Significant at *p* < 0.05

*Abbreviations*: *RDW* Red cell distribution width, *NLR* Neutrophil-to-lymphocyte ratio, *PLR* Platelet-to-lymphocyte ratio, *HGB* Hemoglobin, *Alb* Albumin, *hsCRP* High-sensitivity C-reactive protein

**Table 4:** Multivariate linear regression analysis between PHQ-9 score and NLR

Item	Model 1		Model 2	
	β (95% CI)	*P*	β (95% CI)	*P*
NLR	0.252 (0.194–0.803)	0.001	0.246 (0.175–0.799)	0.002
Alb	− 0.145 (− 0.364–0.011)	0.065	− 0.159 (− 0.397–0.008)	0.059

^*^Significant at *p* < 0.05

Model 1: unadjusted

Model 2: adjusted for age, sex, Kt/V, dialysis duration, history of kidney transplantation, history of hypertension, and Charlson comorbidity index score

*Abbreviations*: *NLR* Neutrophil-to-lymphocyte ratio, *Alb* Albumin, *CI* Confidence interval

## Discussion

We found that the proportion of MHD patients with depressive symptoms was 36.7%, which is similar to the finding of Palmer et al. [2]. We also evaluated the prognostic value of NLR for depressive symptoms risk factors in MHD patients and revealed that NLR was an independent predictor of depressive symptoms in MHD patients.

NLR has been shown to predict death, myocardial infarction, and coronary artery disease [18–20]. Furthermore, numerous epidemiological studies have shown that chronic low-grade inflammation, as measured by NLR, is linked to risk factors such as diabetes mellitus, hypertension, metabolic syndrome, obesity, and hyperlipidemia [21]. In addition, an increased NLR level has been reported to be associated with preoperative depression in patients with gastric cancer [22] and post-stroke depression [13]. NLR may also be a trait marker for suicidal vulnerability in patients with major depression [23]. Gonca et al. [12] found that the NLR level of adolescents with depression is significantly higher than that of healthy controls and is positively correlated with the severity of depression.. In a recent meta-analysis, Mazza et al. [13] found higher NLR levels in patients with major depressive disorder than in healthy controls. In the present study, we found that NLR was an independent predictor of depressive symptoms in MHD patients, which offers new insights and research directions in this area.

NLR is a biomarker that integrates two subtypes of white blood cells (WBCs) that represent two inversely related immune pathways. It is easily calculated using differential WBC counts and is a more stable measurement than individual WBC counts [24]. The association between NLR and depressive symptoms may be explained by several mechanisms. It is well established that both neutrophilia and lymphocytopenia are typical inflammatory responses to various stressful insults. Lymphocytopenia has been confirmed as a marker of poor nutritional condition in MHD patients [25]. Moreover, the prognostic ability of NLR in MHD patients is assumed to rely on the relationship between inflammation and nutritional status [26]. Thus, previous evidence for an association between inflammation [27] and malnutrition [28] and depression in MHD patients may explain the prognostic ability of NLR in predicting depressive symptoms in this population.

Previous studies have demonstrated the role of inflammation in the pathogenesis of depression; for example, inflammation in patients with somatic diseases increases the risk of developing depression. Patients with depression have elevated levels of peripheral and central proinflammatory cytokines, and proinflammatory agents have been shown to facilitate the progression of depressive symptoms [29]. Importantly, the activation of neutrophils can cause oxidative stress by releasing reactive oxygen species, which may contribute to the pathogenesis of depression [30]. Therefore, NLR appears to be a reliable and stable indicator of depressive symptoms in MHD patients.

During inflammation and infection, proinflammatory cytokines may affect erythrocyte maturation by interfering with erythropoietin, which leads to an increase in RDW [31, 32]. Thus, RDW is also considered an inflammatory marker. A population-based cohort study in Iran found that people with severe depressive symptoms had higher RDW than those without depressive symptoms [33]. Demircan et al. [34] reported that RDW and NLR are higher in patients with major depressive disorder than in healthy controls, but these levels decreased in patients following treatment with a selective serotonin reuptake inhibitor. This suggests that RDW and NLR levels are not only useful as biomarkers for diagnosing depression but also for evaluating treatment efficacy. In the present study, we also found that the RDW of MHD patients with mild and moderate/moderately severe depressive symptoms was higher than that of patients with no depressive symptoms. However, the multivariate logistic regression analysis revealed that RDW may not be an independent risk factor for moderate/moderately severe depressive symptoms. We speculate that anemia is a common complication in patients with CKD, especially those on dialysis, in whom the diagnostic value of RDW is not specific. Alb is a common indicator of malnutrition and inflammatory state [35]. We found that the Alb level in MHD patients with moderate/moderately severe depressive symptoms was lower than that in other groups. Moreover, PHQ-9 scores were negatively correlated with Alb level, which is consistent with the report of Huang et al. [36]. Furthermore, Gregg et al. found that low Alb level is associated with progression of depression in a meta-analysis of 34 studies, which included 5652 patients with CKD and ESRD [37]. However, whether depressive symptoms inhibit appetite and lead to malnutrition remains unclear, and the specific mechanisms involved require further study.

Notably, we found that hsCRP was not associated with the PHQ-9 score, which was similar to the report by Joseph et al. who also found no correlation between high hsCRP and increased PHQ-9 score (≥ 10). Given the crossover in symptoms between depression and advanced CKD, it is likely that patients with a more advanced illness who have higher hsCRP values would self-report more depressive symptoms [38]. However, because of missing hsCRP data in 33 patients, we were not able to ascertain the relationship between hsCRP and PHQ-9 score.

Our study has several limitations. First, this was an observational study; therefore, causal relationships between the risk factors and the outcome variable could not be explored. Second, because of the nature of single-center cross-sectional study designs, our study had regional and time limitations. Thus, the results may not accurately reflect the general population. Third, the PHQ-9 is a self-report scale, which assesses depressive symptoms rather than clinical depression. Finally, although we included demographic and laboratory data, we did not assess the impact of socioeconomic and psychological factors.

In summary, we demonstrated that there is a high prevalence of depressive symptoms in MHD patients. NLR, which is an accessible and inexpensive measure, may be a novel biomarker for predicting the presence of depressive symptoms in MHD patients.

## Data Availability

All data generated or analysed during this study are included in this published article.

## Abbreviations

Alb: Albumin
BUN: Blood Urea Nitrogen
CHO: Total Cholesterol
CKD: Chronic Kidney Disease
ESRD: End-stage Renal Disease
HGB: Hemoglobin
MHD: Maintenance Hemodialysis
NLR: Neutrophil-to-lymphocyte Ratio
PHQ-9: Patient Health Questionnaire-9
RDW: Red Cell Distribution Width
Scr: Serum Creatinine
TG: Triglyceride
WBC: White Blood Cell
